# Analysis of Viral Blips Through Week 192 of the DRIVE-FORWARD and DRIVE-AHEAD Phase 3 Studies of Doravirine-Based Regimens in Adults Living With Previously Untreated HIV-1

**DOI:** 10.1093/ofid/ofag258

**Published:** 2026-06-10

**Authors:** Jean-Michel Molina, Chloe Orkin, Roger Paredes, Zhi Jin Xu, Feng-Hsiu Su, Ernest Asante-Appiah, Rebeca M Plank, Rima Lahoulou

**Affiliations:** St-Louis and Lariboisière Hospitals, APHP, University of Paris Cité, Paris, France; SHARE Collaborative, Blizard Institute, Queen Mary University of London, London, UK; Hospital Germans Trias i Pujol, Badalona and Universitat Politècnica de Catalunya–Barcelona HealthTech, Terrassa, Spain; MRL, Merck & Co., Inc., Rahway, New Jersey, USA; MRL, Merck & Co., Inc., Rahway, New Jersey, USA; MRL, Merck & Co., Inc., Rahway, New Jersey, USA; MRL, Merck & Co., Inc., Rahway, New Jersey, USA; MRL, Merck & Co., Inc., Rahway, New Jersey, USA

**Keywords:** blip, doravirine, HIV, nonnucleoside reverse transcriptase inhibitor, transient viremia

## Abstract

**Background:**

We present a post hoc analysis of viral blips (HIV-1 RNA ≥50 copies/mL between 2 values of <50 copies/mL) in the DRIVE-FORWARD and DRIVE-AHEAD doravirine (DOR) Phase 3 studies.

**Methods:**

Adults living with untreated HIV-1 were randomized (1:1) to a DOR-containing regimen or comparator (darunavir/ritonavir- or efavirenz-containing), each with 2 nucleos(t)ide reverse transcriptase inhibitors, for 96 weeks (double-blind base), followed by 96 weeks of a DOR-containing regimen (open-label extension). Cox proportional hazards models were used to analyze relationships between frequency of blips, baseline characteristics, and virologic rebound (2 consecutive HIV-1 RNA ≥50 copies/mL).

**Results:**

In the base studies, blips occurred in 11.1%–11.9% of participants who received DOR and 12.1%–15.4% who received comparator. In the extensions, blips occurred in 5.5%–7.9% of participants. Baseline HIV-1 RNA ≤100 000 copies/mL was associated with lower likelihood of blips in the base studies (hazard ratio [HR], 0.41; [95% CI, 0.29–0.58]) and in participants who continued DOR in the extensions (0.41 [0.19–0.87]). Baseline CD4+ T-cell count ≤200 cells/mm^3^ was associated with increased likelihood of blips in participants who switched to DOR (3.47 [1.43–8.43]). Virologic rebound occurred in 7.0% of participants in the base studies and 3.8% in the extensions. Virologic rebound was more common in participants with blips versus no blips in the base studies (13.6% vs 6.1%; 3.79 [2.32–6.18]).

**Conclusions:**

Blips were associated with increased risk of virologic rebound during the base study, and blips were less likely in participants with lower baseline HIV-1 RNA. Doravirine and comparator groups had similar rates of viral blips.

**Clinical Trials Registration.**  ClinicalTrials.gov NCT02275780, NCT02403674

## Teaser

In 2 Phase 3 studies of adults living with HIV-1, rates of viral blips were similar for doravirine-containing and comparator regimens. Blips were associated with higher baseline HIV-1 RNA, lower baseline CD4+ T-cell counts, and increased risk of virologic rebound.

In 2024, approximately 40.8 million people were living with HIV globally; 77% were accessing antiretroviral therapy (ART), and 73% of people living with HIV were virologically suppressed [1]. Currently, recommended ART for first-line treatment of HIV-1 typically consists of a 2-drug or 3-drug regimen containing drugs from at least 2 distinct mechanistic classes, typically, a nonnucleoside reverse transcriptase inhibitor (NNRTI) or integrase strand transfer inhibitor, and 2 nucleos(t)ide reverse transcriptase inhibitors (NRTIs) [2, 3]. Even when ART successfully suppresses viral load below detection limits, individuals can still experience occasional transient increases in plasma HIV-1 RNA levels [4–8]. These transient increases, hereafter referred to as “blips,” are defined by the US Department of Health and Human Services (DHHS) as “a temporary, detectable increase in the amount of HIV in the blood (viral load) that occurs after ART has effectively suppressed the virus to an undetectable level,” although no exact lower limit of HIV-1 RNA detection is specified [9].

Due to different blip definitions in clinical studies and different assays used to determine HIV-1 RNA levels, the reported incidence of viral blips varies greatly in people who receive ART [10, 11]. Viral blips may occur more frequently in participants who have more advanced HIV-1 infection before starting ART, larger viral reservoirs, or ongoing viral replication because of suboptimal ART adherence [12]. There is conflicting evidence regarding an association between viral blip occurrence and subsequent virologic failure [7, 10, 13, 14]; partly due to the different definitions used across studies for viral blip and virologic failure [3, 15]. As there is no standard approach, viral blips can lead to increased healthcare costs and psychological burden for people living with HIV because of additional outpatient visits, additional viral load testing, and increased monitoring [3, 8, 15, 16].

Doravirine (DOR) is a next-generation NNRTI approved for use in combination with other antiretroviral agents for the treatment of HIV-1 in adults and adolescents [17, 18]. In 2 Phase 3 studies of adults living with previously untreated HIV-1 (DRIVE-FORWARD and DRIVE-AHEAD), DOR-based regimens demonstrated noninferior efficacy and a favorable safety profile compared with ritonavir-boosted darunavir (DRV/r)- and efavirenz (EFV)-based regimens, respectively, through Week 48 [19, 20] and Week 96 [21, 22]. In these studies, DOR-based regimens demonstrated superior lipid profiles compared with DRV/r- and EFV-based regimens, as assessed by low-density and non-high-density lipoprotein cholesterol, and significantly fewer neuropsychiatric events were observed in a DOR-based regimen compared with an EFV-based regimen [21–23]. Furthermore, development of resistance mutations was low in DOR-based regimens (<1% of participants) and common resistance mutations observed in treatment failures with other NNRTIs (ie, K103N and Y181C or E138K) were not observed [21–23]. Open-label extensions of these studies, in which participants continued DOR-based therapy from the base study or switched to a DOR-based regimen at Week 96 through Week 192, demonstrated maintenance of favorable efficacy and safety with DOR-based regimens [24]. The current post hoc analysis of the DRIVE-FORWARD and DRIVE-AHEAD studies assessed the occurrence of viral blips, the possible predictors of viral blips, and the impact of viral blips on subsequent virologic rebound.

## METHODS

### Study Design

DRIVE-FORWARD (MK-1439-018 [P018], NCT02275780) and DRIVE-AHEAD (MK-1439A-021 [P021], NCT02403674) were randomized, double-blind, active-controlled, noninferiority studies in adults living with previously untreated HIV-1 [19–22, 24]. In the DRIVE-FORWARD base study, participants were randomly assigned (1:1) to receive either DOR or DRV/r and 2 investigator-selected NRTIs. In the DRIVE-AHEAD base study, participants were randomly assigned (1:1) to receive a daily fixed-dose tablet of either DOR/lamivudine (3TC)/tenofovir disoproxil fumarate (TDF) (100/300/300 mg) or EFV/emtricitabine (FTC)/TDF (600/200/300 mg) for 96 weeks. After 96 weeks of double-blinded treatment, eligible participants could enter an open-label extension study (Week 96 to Week 192) and either continue a DOR-based regimen (if originally assigned to the DOR group) or switch to the DOR-based regimen (if originally assigned to the comparator group).

### Assessments and Statistical Analysis

The current analysis included participants who had achieved initial HIV-1 RNA suppression to <50 copies/mL and had HIV-1 RNA measured between the date of initial suppression and the date of the last available visit. The main endpoints of interest were viral blip and virologic rebound. Blood plasma was collected for HIV-1 RNA quantification, using the Abbott Laboratories RealTime HIV-1 Viral Load assay [25], on Day 1; at Weeks 4, 8, 16, 24; then every 12 weeks until Week 96 in the base studies; and every 16 weeks for the duration of the extension studies. Viral blip was defined as a measurement of HIV-1 RNA ≥50 copies/mL that was preceded by <50 copies/mL at the previous visit and followed by <50 copies/mL at the next visit. Virologic rebound was defined as 2 consecutive measures of HIV-1 RNA ≥50 copies/mL at least 1 week apart at any time during the study after an initial HIV-1 RNA suppression of <50 copies/mL. Participants with viral rebound were discontinued from both studies. Two main time periods were assessed: the double-blind base study, Day 1 to Week 96, and the open-label extension study, Week 96 to Week 192. For the double-blind base studies, the incidence of blips was also analyzed from Day 1 to Week 48 and from Week 48 to Week 96.

Data from DOR-based regimens (DOR and 2 NRTIs, or DOR/3TC/TDF) and comparator regimens (DRV/r and 2 NRTIs, or EFV/FTC/TDF) were assessed per study for descriptive statistics or pooled to analyze the overall relationship between baseline characteristics and viral blips or viral rebound. A Cox proportional hazards model was used to analyze the relationship between viral blips and baseline characteristic factors, and a Cox model with time-varying blip status was used to analyze the relationship between blips and virologic rebound. The covariates used in the model were age group (<50 or ≥50 years of age), history of AIDS (yes or no), baseline CD4+ T-cell count (≥200 or <200 cells/mm^3^), baseline HIV-1 RNA (≤100 000 or >100 000 copies/mL), race (Black, White, or other), sex (male or female), study (P018 or P021), treatment group, and interaction of study and treatment group.

## RESULTS

### Double-Blind Base Studies (Day 1 to Week 96)

Among a total of 1494 participants who were treated, 1338 (89.6%) met the criteria for this analysis: 678 who had been originally assigned to the DOR-based regimens and 660 who had been assigned to the comparator. A total of 109 participants (7.3%) did not achieve an initial response of HIV-1 RNA <50 copies/mL and 47 participants (3.1%) had no HIV-1 RNA measurements between the initial response and the last visit and were excluded from the analysis. Blips occurred in 169 of 1338 participants (12.6%) overall: 11.1% and 11.9% of participants in the DOR-based regimens and 15.4% and 12.1% of participants in the comparator regimens in DRIVE-FORWARD and DRIVE-AHEAD, respectively (Table 1). The blip incidence rates were 0.07 per person-year for both DOR-based regimens and 0.10 and 0.07 per person-year for the comparator regimens in DRIVE-FORWARD and DRIVE-AHEAD, respectively. The percentages of HIV-1 RNA measurements which showed blips were 0.92% and 1.22% in the DOR-based regimens and 1.22% and 1.05% in the comparator regimens, in DRIVE-FORWARD and DRIVE-AHEAD, respectively. A summary of baseline characteristics for participants with and without blips is shown in Supplementary Table 1. Blips occurred at similar frequencies in the first half of the double-blind base studies (Day 1 to Week 48) compared with the second half (Week 48 to Week 96) for DOR-based regimens (first half, 5.4%–5.8%; second half, 5.6%–8.3%) and comparator regimens (first half, 6.5%–9.5%; second half, 6.5%–7.1%) (Table 2). In all treatment groups, for most participants with blips only 1 episode was detected (70.0%–92.3%) to Week 96 (Supplementary Figure 1). The median viral load at first blip, and in most cases the only blip event, was low, ranging from 61.5 to 73.0 copies/mL. Among participants with blips in DRIVE-FORWARD, subsequent virologic rebound occurred in 21.1% (n = 8) of participants in DOR-based regimens and in 13.5% (n = 7) in comparator regimens, whereas, in DRIVE-AHEAD, subsequent virologic rebound occurred in 12.5% (n = 5) of participants in DOR-based regimens and in 7.7% (n = 3) in comparator regimens (Table 1). Of the 23 participants who had viral rebound after a blip in the base studies, 11 (48%) had a viral load <200 copies/mL. When the data were pooled, the proportion of participants with virologic rebound was higher among participants with viral blips (23 of 169 [13.6%]) compared with participants who did not have blips (71 of 1169 [6.1%]).

**Table 1.** Summary of Viral Blips in DRIVE-FORWARD and DRIVE-AHEAD During the Double-Blind Base Studies (Day 1 to Week 96) and Extension Studies (Week 96 to Week 192) by Treatment Group

	Day 1 to Week 96				Week 96 to Week 192			
	DRIVE-FORWARD		DRIVE-AHEAD		DRIVE-FORWARD		DRIVE-AHEAD	
	DOR + 2 NRTIs	DRV/r + 2 NRTIs	DOR/3TC/TDF	EFV/FTC/TDF	DOR continued (DOR + 2 NRTIs)	DOR switched (DOR + 2 NRTIs)	DOR continued (DOR/3TC/TDF)	DOR switched (DOR/3TC/TDF)
Participants, N	342	338	336	322	253	225	286	265
Total # of blips	42	56	54	46	14	22	20	24
Participants who had blips n (%)^a^	38 (11.1)	52 (15.4)	40 (11.9)	39 (12.1)	14 (5.5)	17 (7.6)	19 (6.6)	21 (7.9)
1 blip^b^	35 (92.1)	48 (92.3)	28 (70.0)	35 (89.7)	14 (100.0)	13 (76.5)	18 (94.7)	18 (85.7)
2 blips^b^	2 (5.3)	4 (7.7)	10 (25.0)	3 (7.7)	0	3 (17.6)	1 (5.3)	3 (14.3)
3 blips^b^	1 (2.6)	0	2 (5.0)	0	0	1 (5.9)	0	0
≥4 blips^b^	0	0	0	1 (2.6)	0	0	0	0
VL at first blip, median (IQR), copies/mL	72.5 (56.0–120.0)	70.5 (58.0–92.0)	61.5 (55.0–196.5)	73.0 (56.0–98.0)	66.0 (63.0–76.0)	85.0 (57.0–126.0)	74.0 (56.0–154.0)	76.0 (61.0–89.0)
Participants with virologic rebound after blip, n (%)^b^	8 (21.1)	7 (13.5)	5 (12.5)	3 (7.7)	1 (7.1)	1 (5.9)	1 (5.3)	3 (14.3)
VL <200 copies/mL^b^	3 (7.9)	3 (5.8)	3 (7.5)	2 (5.1)	1 (7.1)	1 (5.9)	1 (5.3)	2 (9.5)
VL ≥200 copies/mL^b^	5 (13.2)	4 (7.7)	2 (5.0)	1 (2.6)	0	0	0	1 (4.8)

Abbreviations: 3TC, lamivudine; DOR, doravirine; EFV, efavirenz; FTC, emtricitabine; IQR, interquartile range; NRTI, nucleos(t)ide reverse transcriptase inhibitor; TDF, tenofovir disoproxil fumarate; VL, viral load.

^a^Denominator for percentage is the number of participants included in the analysis.

^b^Denominator for percentage is the number of participants who had blips.

**Table 2.** Summary of Viral Blips in DRIVE-FORWARD and DRIVE-AHEAD During the First Half (Day 1 to Week 48) and Second Half (Week 48 to Week 96) of the Double-Blind Base Studies by Treatment Group

	DRIVE-FORWARD				DRIVE-AHEAD			
	DOR + 2 NRTIs N = 342		DRV/r + 2 NRTIs N = 338		DOR/3TC/TDF N = 336		EFV/FTC/TDF N = 322	
	Day 1 to Week 48	Week 48 to Week 96	Day 1 to Week 48	Week 48 to Week 96	Day 1 to Week 48	Week 48 to Week 96	Day 1 to Week 48	Week 48 to Week 96
Total # of blips	20	22	32	24	20	34	22	24
Participants who had blips, n (%)^a^	20 (5.8)	19 (5.6)	32 (9.5)	24 (7.1)	18 (5.4)	28 (8.3)	21 (6.5)	21 (6.5)
1 blip^b^	20 (100.0)	17 (89.5)	32 (100.0)	24 (100.0)	16 (88.9)	22 (78.6)	20 (95.2)	19 (90.5)
2 blips^b^	0	1 (5.3)	0	0	2 (11.1)	6 (21.4)	1 (4.8)	1 (4.8)
3 blips^b^	0	1 (5.3)	0	0	0	0	0	1 (4.8)
≥4 blips^b^	0	0	0	0	0	0	0	0

Abbreviations: 3TC, lamivudine; DOR, doravirine; EFV, efavirenz; FTC, emtricitabine; NRTI, nucleos(t)ide reverse transcriptase inhibitor; TDF, tenofovir disoproxil fumarate.

^a^Denominator for percentage is the number of participants included in the analysis.

^b^Denominator for percentage is the number of participants who had blips.

In the Cox proportional hazards model of pooled data for all treatment groups, risk for blips was significantly lower in participants with baseline HIV-1 RNA ≤100 000 copies/mL than in those with baseline HIV-1 RNA >100 000 copies/mL (hazard ratio [HR], 0.41; 95% CI, 0.29–0.58; *P* < 0.001) (Table 3). Treatment regimen was not significantly associated with risk for blips (Table 3). Virologic rebound occurred in 94 of 1338 participants (7.0%) in the base studies. The risk of virologic rebound was significantly higher in participants who had blips than in those who did not (HR, 3.79; 95% CI, 2.32–6.18; *P* < 0.001) (Table 4). Additionally, the risk of virologic rebound was significantly higher in the group aged <50 years than in the group ≥50 years (HR, 4.02; 95% CI, 1.27–12.76; *P* = 0.018) and was significantly higher for the P018 comparator (DRV/r and 2 NRTIs) than for the P021 comparator (EFV/FTC/TDF) (HR, 2.00; 95% CI, 1.05–3.80; *P* = 0.035). Both treatment regimens were comparable, and neither was significantly associated with risk of virologic rebound after blip (Table 4).

**Table 3.** Hazard Ratio for Blips in DRIVE-FORWARD and DRIVE-AHEAD Studies, Day 1 to Week 96, Pooled Data (All Treatment Groups)

Covariates	Hazard Ratio (95% CI)	*P* Value
Baseline characteristic		
Age group (<50 vs ≥50 y)	0.80 (0.51–1.26)	0.344
History of AIDS (yes vs no)	1.01 (0.59–1.74)	0.957
Baseline CD4+ T-cell count (≤200 vs >200 cells/mm^3^)	1.08 (0.65–1.80)	0.754
Baseline HIV-1 RNA (≤100 000 vs >100 000 copies/mL)	0.41 (0.29–0.58)	<0.001
Race group: Black vs White	1.55 (1.05–2.27)	0.027
Race group: Other vs White	1.10 (0.70–1.71)	0.678
Sex group (female vs male)	0.73 (0.46–1.17)	0.192
Study and treatment group		
P018: DOR + 2 NRTIs vs DRV/r + 2 NRTIs	0.66 (0.44–1.01)	0.057
P021: DOR/3TC/TDF vs EFV/FTC/TDF	0.96 (0.62–1.50)	0.865
DOR + 2 NRTIs (P018) vs DOR/3TC/TDF (P021)	0.92 (0.58–1.46)	0.730
DRV/r + 2 NRTIs (P018) vs EFV/FTC/TDF (P021)	1.34 (0.86–2.08)	0.201

Abbreviations: 3TC, lamivudine; DOR, doravirine; DRV/r, ritonavir-boosted darunavir; EFV, efavirenz; FTC, emtricitabine; NRTI, nucleos(t)ide reverse transcriptase inhibitor; TDF, tenofovir disoproxil fumarate.

**Table 4.** Hazard Ratio for Virologic Rebound in DRIVE-FORWARD and DRIVE-AHEAD, Day 1 to Week 96, Pooled Data (All Treatment Groups)

Covariates	Hazard Ratio (95% CI)	*P* Value
Baseline characteristic		
Blip status (yes vs no)	3.79 (2.32–6.18)	<0.001
Age group (<50 vs ≥50 y)	4.02 (1.27–12.76)	0.018
History of AIDS (yes vs no)	1.03 (0.50–2.12)	0.927
Baseline CD4+ T-cell count (≤200 vs >200 cells/mm^3^)	1.45 (0.75–2.81)	0.265
Baseline HIV-1 RNA (≤100 000 vs >100 000 copies/mL)	0.67 (0.41–1.10)	0.115
Race group: Black vs White	1.24 (0.74–2.11)	0.416
Race group: Other vs White	1.37 (0.77–2.46)	0.287
Sex group (female vs male)	1.57 (0.93–2.67)	0.094
Study and treatment group		
P018: DOR + 2 NRTIs vs DRV/r + 2 NRTIs	0.74 (0.42–1.30)	0.296
P021: DOR/3TC/TDF vs EFV/FTC/TDF	1.06 (0.58–1.94)	0.840
DOR + 2 NRTIs (P018) vs DOR/3TC/TDF (P021)	1.38 (0.72–2.67)	0.332
DRV/r + 2 NRTIs (P018) vs EFV/FTC/TDF (P021)	2.00 (1.05–3.80)	0.035

Abbreviations: 3TC, lamivudine; DOR, doravirine; DRV/r, ritonavir-boosted darunavir; EFV, efavirenz; FTC, emtricitabine; NRTI, nucleos(t)ide reverse transcriptase inhibitor; TDF, tenofovir disoproxil fumarate.

### Open-Label Extensions (Week 96 to Week 192)

Of 1052 participants who entered the study extensions, 1029 (97.8%) met the criteria for the analysis: 539 who continued the DOR-containing regimen (“DOR continued” participants) and 490 who switched from a comparator to a DOR-containing regimen (“DOR switched” participants). A total of 23 participants (2.2%) were excluded because they had no HIV-1 RNA measurements on or after the first visit of the study extension. Blips were less common during the study extensions than during the base studies, occurring in 71 of 1029 participants (6.9%) overall. In DRIVE-FORWARD, blips occurred in 5.5% of DOR continued participants and in 7.6% of DOR switched participants. Blips occurred at a similar frequency in DRIVE-AHEAD: in 6.6% of DOR continued participants and 7.9% of DOR switched participants (Table 1). The incidence rates for blips were 0.03 and 0.04 per person-year for DOR-based regimens and 0.05 per person-year for both comparator regimens in DRIVE-FORWARD and DRIVE-AHEAD, respectively. The percentages of HIV-1 RNA measurements that showed blips were 0.30% and 0.42% in the DOR-based regimens and 0.60% and 0.61% in the comparator regimens, in DRIVE-FORWARD and DRIVE-AHEAD, respectively. Baseline characteristics for participants who had blips in the study extensions and those who did not are summarized in Supplementary Table 1. Most participants who had blips had only 1 episode (Supplementary Figure 1), and the median viral load at first blip was low, ranging from 66.0 to 85.0 copies/mL.

Base study baseline HIV-1 RNA ≤100 000 copies/mL was associated with lower risk of blips in DOR continued participants (HR, 0.41; 95% CI, 0.19–0.87; *P* = 0.021; Table 5). A base study baseline CD4+ T-cell count ≤200 cells/mm^3^ was associated with increased risk of blips in DOR switched participants (HR, 3.47; 95% CI, 1.43–8.43; *P* = 0.006; Table 5). Virologic rebound occurred in 39 of 1029 participants (3.8%) in the study extensions and was numerically higher among participants who had blips (6 of 71 [8.5%]) than among those who did not have blips (33 of 958 [3.4%]); however, the impact of blips on virologic rebound in the study extensions was unclear due to the low number of events. Of the 6 participants who had virologic rebound after a blip, 5 had a viral load <200 copies/mL (Table 1).

**Table 5.** Hazard Ratio for Blips in DRIVE-FORWARD and DRIVE-AHEAD Study Extensions, Week 96 to Week 192, DOR Continued and Switched Groups

Covariates	DOR Continued		DOR Switched	
	Hazard Ratio (95% CI)	*P* Value	Hazard Ratio (95% CI)	*P* Value
Baseline characteristic				
Age group (<50 vs ≥50 y)	1.14 (0.39–3.35)	0.806	1.13 (0.39–3.31)	0.818
History of AIDS (yes vs no)	1.61 (0.54–4.81)	0.396	1.82 (0.68–4.89)	0.234
Baseline CD4+ T-cell count (≤200 vs >200 cells/mm^3^)	1.10 (0.34–3.59)	0.873	3.47 (1.43–8.43)	0.006
Baseline HIV-1 RNA (≤100 000 vs >100 000 copies/mL)	0.41 (0.19–0.87)	0.021	1.42 (0.61–3.29)	0.414
Race group: Black vs White	0.98 (0.36–2.70)	0.968	0.70 (0.25–1.96)	0.495
Race group: Other vs White	0.60 (0.23–1.61)	0.314	2.27 (0.98–5.29)	0.057
Sex group (female vs male)	0.29 (0.07–1.29)	0.105	1.13 (0.45–2.83)	0.791

Abbreviation: DOR, doravirine.

## DISCUSSION

In the DRIVE-AHEAD and DRIVE-FORWARD studies, DOR-containing regimens were efficacious and well tolerated in adults living with previously untreated HIV-1. There is clinical interest in understanding the significance of viral blips and how increased risk of blips may be associated with certain clinical characteristics, treatment regimens, and subsequent viral rebound or treatment failure. Due to the intense monitoring of participants in the clinical study setting, these 2 studies provided an opportunity to investigate the timing and number of blips, the association of blips with DOR-based or comparator treatment regimens, participant clinical characteristics, and subsequent viral rebound. In the DRIVE-FORWARD and DRIVE-AHEAD base studies, the incidence of viral blips was similar in participants who received a DOR-containing regimen and those who received a comparator regimen. In the study extensions, the incidence of viral blips was lower than in the base studies and was similar in participants who continued or switched to a DOR-containing regimen. Most participants who had viral blips had a single occurrence, and the risk of blips was not associated with the specific treatment received.

Overall, 12.6% of participants had viral blips in the base studies, whereas 6.9% of participants had viral blips in the extension study. These incidence rates align with observed rates of viral blips in other studies, although the reported incidence rates vary widely from 10% to 50% [4, 7, 8, 10]. The definitions of viral blip and virologic rebound may differ from study to study, which could be a reason for the large variability in reported incidence [7, 10, 11]. In the current analysis, our definition of viral blip aligned with that used by the DHHS and was driven by the lower limit of detection of the quantification assay used (Abbott RealTime HIV-1, 40 copies/mL) [15, 25]. Assay type has also been shown to affect the detection of viral blips [10], which makes cross-study comparisons difficult.

A baseline HIV-1 RNA ≤100 000 copies/mL was associated with lower risk of viral blips in all treatment groups during the base studies and in the DOR continued groups during the study extensions. A baseline CD4+ T-cell count ≤200 cells/mm^3^ was associated with higher risk of viral blips in the DOR switch groups during the study extensions. In an unrelated study, a Swedish cohort of adults living with untreated HIV-1 who received first-line ART, baseline viral load was significantly higher in those who subsequently experienced viral blips [10]. In another study, pretreatment CD4+ T-cell counts were significantly lower in those participants who had viral blips and low-level viremia than in those with viral suppression [12, 26]. It is unknown why low CD4+ T-cell count was associated with a higher risk of viral blips in the extension study for DOR switch groups only, and this could be explored in future studies.

The incidence of virologic rebound was low overall: 7.0% during the base studies and 3.8% during the study extensions. In the base studies, viral blips were associated with increased risk of virologic rebound. In some studies, viral blips were associated with virologic rebound and virologic failure, but not in others [5, 7, 8, 12]. In the current analysis, most participants in the base studies had 1 blip (70.0%–92.3%), and the median viral load at first blip was low (61.5–73.0 copies/mL). The risk of virologic failure was demonstrated by others to be higher with incidence of viral blips <200 copies/mL and with blips of 50–500 copies/mL, compared with no blips [10, 12].

In the study extensions, 5 of the 6 participants with virologic rebound after a blip had low-level viremia (rebound viral load <200 copies/mL). Persistent, low-level viremia of 50–200 copies/mL has been associated with an increased risk of virologic failure [11, 27]. The participants in the current studies discontinued after virologic rebound, and longer term virologic outcomes are unknown.

In a previous analysis of DRIVE-AHEAD and DRIVE-FORWARD, mutational resistance to DOR and NRTIs was evaluated in participants who met the criteria for resistance testing: 2 consecutive measures ≥1 week apart of HIV-1 RNA ≥200 copies/mL at Week 24 or Week 36; ≥50 copies/mL at Week 48 or at any time during the study after initial response of <50 copies/mL, or early discontinuation of study treatment [24]. A low proportion of participants had resistance to DOR: 2% of participants treated with DOR-containing regimens in the base studies and 1% of participants in the study extensions [24]. Similarly, a low proportion of participants had resistance to NRTIs: 1% of participants treated with DOR-containing regimens in the base studies and <1% of participants in the extension studies [24]. Further analysis is necessary to understand whether mutational resistance is associated with an increased risk of viral blips or virologic rebound.

The full clinical implications of viral blips are unclear, but considerations include clinical management of the individual being treated, as well as implications for public health. The observation of blips may be an indication of the need for renewed adherence counseling or other support. Furthermore, the European AIDS Clinical Society guidelines recommend that for viral load >50 copies/mL, viral load should be checked again in 1 or 2 months, and if viral load is confirmed at >200 copies/mL, a decision can be made to switch treatment regimens, considering other additional factors [3]. In the current study, specific treatment regimen had no association with risk of blips. With regard to implications for the undetectable = untransmittable (U = U) strategy for HIV prevention, the DHHS recommends that if a person living with HIV receiving ART has a viral load of ≥200 copies/mL, another form of prevention should be used to protect against HIV transmission until a viral load of <200 copies/mL is achieved [15, 28].

The frequency of viral load testing should be considered when interpreting these results and comparing other studies that examined rates of viral blips. Testing for HIV-1 viral load occurred 7 times in the first year of the base study and, subsequently, every 3 to 4 months for the duration of the base and extension studies over a period of almost 4 years. Participants initiating ART are more closely monitored in the first year of treatment until viral load is suppressed, so this may reflect a typical sampling frequency in treatment-naive participants [10, 15]. For participants on a stable antiretroviral regimen, viral load measurements are recommended every 3 to 4 months to confirm continuous viral suppression, and every 6 months thereafter if participants have a stable clinical and immunologic status and are not at risk for inadequate adherence [3, 15]. The more frequent viral load monitoring in the current studies compared with what is recommended in routine clinical practice may have led to increased blip detection compared with real-world care. However, the frequency of viral monitoring still may not have captured all viral blips that occurred, and some correlations between the frequency of blips, baseline characteristics, and virologic rebound may have been missed.

For a post hoc analysis, these findings may not be readily generalizable to the broader population of adults living with previously untreated HIV-1. The sample sizes of participants who had viral blips and viral rebound were small, particularly in the extension study, which might have limited the ability to make correlations with baseline characteristics. Furthermore, the definition of virologic rebound used—2 consecutive measures of HIV-1 RNA ≥50 copies/mL at least 1 week apart, which led to study discontinuation—likely does not reflect real-world clinical practice, in which further viral load monitoring would occur in parallel to continuation of treatment. In the current studies, most participants were male, which might limit the application of results to women living with HIV.

## CONCLUSION

In this post hoc analysis of the DRIVE-AHEAD and DRIVE-FORWARD studies in adults living with previously untreated HIV-1, viral blips increased the likelihood of subsequent virologic rebound during the base studies. Viral blips were less likely to occur in participants with baseline HIV-1 RNA ≤100 000 copies/mL in the base and in participants who continued DOR-based therapy in the extension studies, and were more likely with baseline CD4+ T-cell count ≤200 cells/mm^3^ in participants who switched to DOR in the extension studies. Rates of viral blips were similar in the DOR-based and comparator regimens and decreased in the extension study.

## Supplementary Material

ofag258_Supplementary_Data

## Supplementary Data

Supplementary materials are available at *Open Forum Infectious Diseases* online. Consisting of data provided by the authors to benefit the reader, the posted materials are not copyedited and are the sole responsibility of the authors, so questions or comments should be addressed to the corresponding author.
