# Supplementary material for: Analysis of Viral Blips Through Week 192 of the DRIVE-FORWARD and DRIVE-AHEAD Phase 3 Studies of Doravirine-Based Regimens in Adults Living With Previously Untreated HIV-1
Source: Open Forum Infect Dis. 2026 Jun 10;13(6):ofag258. doi: 10.1093/ofid/ofag258 (PMC13251338; doi:10.1093/ofid/ofag258)
Supplement: ofag258_Supplementary_Data [file ofag258_supplementary_data.docx]

**Supplementary Data**

**Supplementary Table 1. Summary of Participant Characteristics by Blip Status Day 1 to Week 96, and Week 96 to Week 192, DOR Continued and DOR Switched Groups**

| **Participant characteristic, n (%)** | **Day 1 to Week 96** | | | | **Week 96 to Week 192** | | | |
| --- | --- | --- | --- | --- | --- | --- | --- | --- |
|  | **DOR + 2 NRTIs (P018) or DOR/3TC/TDF (P021)** | | **DRV/v + 2 NRTIs (P018) or EFV/FTC/TDV (P021)** | | **DOR Continued^a^** | | **DOR Switched^b^** | |
|  | **Blips**  **N = 78** | **No blips**  **N = 600** | **Blips**  **N = 91** | **No blips**  **N = 569** | **Blips**  **N = 33** | **No blips**  **N = 506** | **Blips**  **N = 38** | **No blips**  **N = 452** |
| Male | 66 (84.6) | 503 (83.8) | 81 (89.0) | 486 (85.4) | 31 (93.9) | 417 (82.4) | 32 (84.2) | 390 (86.3) |
| Age, median (range), years | 33.0 (20–65) | 32.0 (18–70) | 33.0 (18–69) | 32.0 (18–69) | 30.0 (19–57) | 33.0 (18–70) | 33.0 (21–69) | 32.0 (18–67) |
| **Race** | | | | | | | | |
| American Indian or Alaska Native | 1 (1.3) | 8 (1.3) | 0 | 9 (1.6) | 1 (3.0) | 6 (1.2) | 1 (2.6) | 5 (1.1) |
| Asian | 5 (6.4) | 61 (10.2) | 13 (14.3) | 51 (9.0) | 4 (12.1) | 53 (10.5) | 9 (23.7) | 43 (9.5) |
| Black or African American | 21 (26.9) | 105 (17.5) | 19 (20.9) | 110 (19.3) | 5 (15.2) | 90 (17.8) | 5 (13.2) | 78 (17.3) |
| Multiple | 8 (10.3) | 46 (7.7) | 4 (4.4) | 46 (8.1) | 1 (3.0) | 43 (8.5) | 2 (5.3) | 43 (9.5) |
| Native Hawaiian or Other Pacific Islander | 0 | 0 | 0 | 2 (0.4) | 0 | 0 | 0 | 2 (0.4) |
| White | 43 (55.1) | 380 (63.3) | 55 (60.4) | 350 (61.5) | 22 (66.7) | 314 (62.1) | 21 (55.3) | 281 (62.2) |
| Missing | 0 | 0 | 0 | 1 (0.2) | 0 | 0 | 0 | 0 |
| **Ethnicity** | | | | | | | | |
| Hispanic or Latino/a | 26 (33.3) | 175 (29.2) | 18 (19.8) | 166 (29.2) | 9 (27.3) | 154 (30.4) | 11 (28.9) | 130 (28.8) |
| Not Hispanic or Latino/a | 52 (66.7) | 419 (69.8) | 71 (78.0) | 392 (68.9) | 24 (72.7) | 349 (69.0) | 27 (71.1) | 315 (69.7) |
| Not reported | 0 | 5 (0.8) | 1 (1.1) | 5 (0.9) | 0 | 3 (0.6) | 0 | 4 (0.9) |
| Unknown | 0 | 1 (0.2) | 1 (1.1) | 6 (1.1) | 0 | 0 | 0 | 3 (0.7) |
| **Region** | | | | | | | | |
| Africa | 10 (12.8) | 42 (7.0) | 6 (6.6) | 35 (6.2) | 0 | 41 (8.1) | 0 | 30 (6.6) |
| Asia-Pacific | 5 (6.4) | 63 (10.5) | 10 (11.0) | 48 (8.4) | 6 (18.2) | 56 (11.1) | 7 (18.4) | 43 (9.5) |
| Europe | 23 (29.5) | 216 (36.0) | 42 (46.2) | 207 (36.4) | 12 (36.4) | 183 (36.2) | 11 (28.9) | 177 (39.2) |
| Latin America | 14 (17.9) | 105 (17.5) | 11 (12.1) | 97 (17.0) | 6 (18.2) | 100 (19.8) | 8 (21.1) | 85 (18.8) |
| North America | 26 (33.3) | 174 (29.0) | 22 (24.2) | 182 (32.0) | 9 (27.3) | 126 (24.9) | 12 (31.6) | 117 (25.9) |
| Baseline HIV-1 RNA, median (range), log_10_ copies/mL^c^ | 4.6 (3.0-6.2) | 4.3 (2.4-6.4) | 4.7 (3.0-6.4) | 4.4 (2.4-6.5) | 4.8 (3.4-5.9) | 4.3 (2.4-6.4) | 4.4 (3.4-6.3) | 4.4 (2.4-6.4) |
| **Stratum NRTTI backbone (P018 only)** | | | | | | | | |
| FTC/TDF | 35 (44.9) | 262 (43.7) | 46 (50.5) | 250 (43.9) | 13 (39.4) | 202 (39.9) | 13 (34.2) | 182 (40.3) |
| ABC/3TC | 3 (3.8) | 42 (7.0) | 6 (6.6) | 36 (6.3) | 1 (3.0) | 37 (7.3) | 4 (10.5) | 26 (5.8) |
| Missing | 40 (51.3) | 296 (49.3) | 39 (42.9) | 283 (49.7) | 19 (57.6) | 267 (52.8) | 21 (55.3) | 244 (54.0) |
| **Baseline viral subtype category** | | | | | | | | |
| Clade B | 53 (67.9) | 399 (66.5) | 49 (53.8) | 414 (72.8) | 24 (72.7) | 327 (64.6) | 23 (60.5) | 312 (69.0) |
| Non–clade B | 25 (32.1) | 200 (33.3) | 42 (46.2) | 155 (27.2) | 9 (27.3) | 178 (35.2) | 15 (39.5) | 140 (31.0) |
| Missing | 0 | 1 (0.2) | 0 | 0 | 0 | 1 (0.2) | 0 | 0 |

Abbreviations: 3TC, lamivudine; ABC, abacavir; DOR, doravirine; DRV/r, ritonavir-boosted darunavir; EFV, efavirenz; FTC, emtricitabine; NRTI, nucleos(t)ide reverse transcriptase inhibitor; NRTTI, nucleoside reverse transcriptase translocation inhibitor; TDF, tenofovir disoproxil fumarate.

^a^Originally assigned to a DOR-containing regimen (DOR + 2 NRTIs or DOR/3TC/TDF) during the period from Day 1 to Week 96.

^b^Originally assigned to a comparator regimen (DRV/r + 2 NRTIs or EFV/3TC/TDF) during the period from Day 1 to Week 96.

^c^One participant missing data in the DOR switched subgroup with blips.

**Supplementary Figure 1.** Number of viral blips in participants who had blips in DRIVE-FORWARD and DRIVE-AHEAD by treatment group. *A,* During the double-blind base studies (Day 1 to Week 96). *B*, During the extension studies (Week 96 to Week 192) by treatment group. Abbreviations: 3TC, lamivudine; DOR, doravirine; DRV/r, ritonavir-boosted darunavir; EFV, efavirenz; FTC, emtricitabine; NRTI, nucleos(t)ide reverse transcriptase inhibitor; TDF, tenofovir disoproxil fumarate.

**
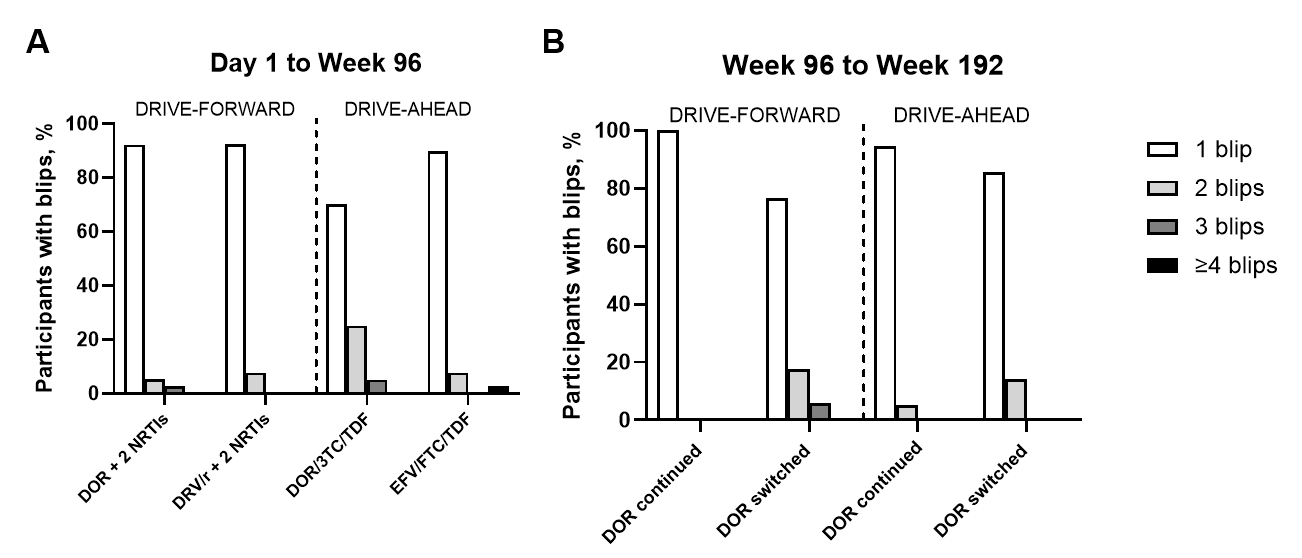
**
